# Dynamic Modelling Reveals ‘Hotspots’ on the Pathway to Enzyme-Substrate Complex Formation

**DOI:** 10.1371/journal.pcbi.1004811

**Published:** 2016-03-11

**Authors:** Shane E. Gordon, Daniel K. Weber, Matthew T. Downton, John Wagner, Matthew A. Perugini

**Affiliations:** 1 Department of Biochemistry and Genetics, La Trobe Institute for Molecular Science, La Trobe University, Melbourne, Victoria, Australia; 2 Computational Biophysics, IBM Research - Australia, Carlton, Victoria, Australia; Max Planck Institute for Biophysical Chemistry, GERMANY

## Abstract

Dihydrodipicolinate synthase (DHDPS) catalyzes the first committed step in the diaminopimelate pathway of bacteria, yielding amino acids required for cell wall and protein biosyntheses. The essentiality of the enzyme to bacteria, coupled with its absence in humans, validates DHDPS as an antibacterial drug target. Conventional drug design efforts have thus far been unsuccessful in identifying potent DHDPS inhibitors. Here, we make use of contemporary molecular dynamics simulation and Markov state models to explore the interactions between DHDPS from the human pathogen *Staphylococcus aureus* and its cognate substrate, pyruvate. Our simulations recover the crystallographic DHDPS-pyruvate complex without *a priori* knowledge of the final bound structure. The highly conserved residue Arg140 was found to have a pivotal role in coordinating the entry of pyruvate into the active site from bulk solvent, consistent with previous kinetic reports, indicating an indirect role for the residue in DHDPS catalysis. A metastable binding intermediate characterized by multiple points of intermolecular interaction between pyruvate and key DHDPS residue Arg140 was found to be a highly conserved feature of the binding trajectory when comparing alternative binding pathways. By means of umbrella sampling we show that these binding intermediates are thermodynamically metastable, consistent with both the available experimental data and the substrate binding model presented in this study. Our results provide insight into an important enzyme-substrate interaction in atomistic detail that offers the potential to be exploited for the discovery of more effective DHDPS inhibitors and, in a broader sense, dynamic protein-drug interactions.

## Author Summary

Interactions between proteins and ligands underpin many important biological processes, such as binding of substrates to their cognate enzymes in the process of catalysis. These interactions are complex, often requiring several intermediate steps to fully transition into the bound state. Here, we have used computational simulation to study binding of pyruvate to Dihydrodipicolinate synthase (DHDPS), an enzyme in the bacterial diaminopimelate pathway. In bacteria, such as the human pathogen *S. aureus*, DHDPS functions to make building blocks necessary for protein and bacterial cell wall biosyntheses. As the enzyme is absent in humans, yet essential for bacterial growth, DHDPS is a valid target for broad-range antibiotics. However, known DHDPS inhibitors show poor potency. One avenue that has not yet been taken into consideration for inhibitor design is the dynamics of DHDPS’s interaction with its reaction substrates (e.g. pyruvate). Using molecular dynamics simulation, we find that pyruvate binding to DHDPS must pass through a transition intermediate ‘hotspot’ in which the substrate is held in place by a dense network of noncovalent bonds. Given that many of the protein residues involved in this interaction are also shared by DHDPS from many pathogenic bacteria, this binding intermediate ‘hotspot’ may help in development of better broad-range DHDPS inhibitors.

## Introduction

**Fig 1:**
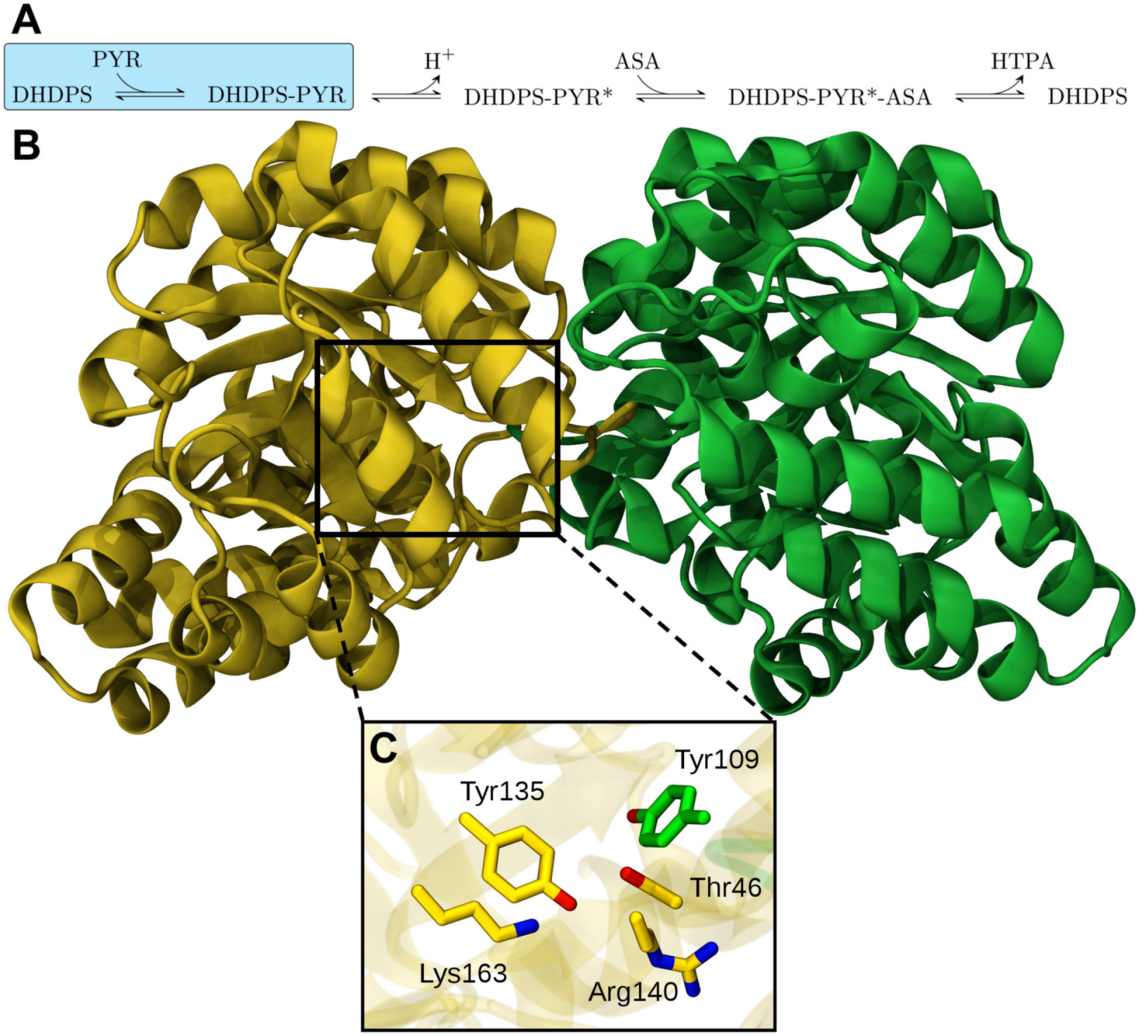
Kinetic mechanism and structure. (A) The DHDPS-catalyzed reaction follows a classic bi-bi substrate model, requiring the first substrate (PYR; pyruvate) bind the enzyme for the second substrate (ASA) to be recruited to the active site and ultimately liberate the reaction product (HTPA). The initial pyruvate-binding portion of the reaction scheme is highlighted in cyan. (B) Quaternary structure of the DHDPS dimer. (C) Licorice representation of key active site residues. Protein chains A and B are shown in yellow and green, respectively.

Dihydrodipicolinate synthase (DHDPS) catalyzes the first and committed step in the diaminopimelate (DAP) biosynthesis pathway of bacteria and plants, namely the condensation of pyruvate and (*S*)-aspartate-*β*-semialdehyde (ASA) to (4*S*)-4-hydroxy-2,3,4,5-tetrahydro-(2*S*)-dipicolinic acid (HTPA) [1–4] (Fig 1A). (4*S*)-4-hydroxy-2,3,4,5-tetrahydro-(2*S*)-dipicolinic acid (HTPA) is then converted via a series of enzyme-catalyzed reactions to yield *meso*-diaminopimelate (*meso*-DAP) and (*S*)-lysine, which are important metabolites for cell wall and protein biosyntheses. Gene knock-out studies demonstrate that *dapA*, which encodes dihydrodipicolinate synthase (DHDPS), is an essential gene [5–9]. Given its essentiality to bacteria and plants, but absence in humans, DHDPS has gained considerable traction as a promising target for both antimicrobial drugs and herbicides [3, 10, 11]. Despite sustained interest in DHDPS, potent inhibitors of the enzyme have not yet been realized [3, 10, 11]. The most effective inhibitors discovered to date are predominantly derived by analogy to HTPA, which nonetheless show poor (low millimolar) inhibitory potency. There is thus a need to consider other factors in the rational design of DHDPS inhibitors. One such factor is the dynamics of the DHDPS-substrate interaction. Herein, we explore this phenomenon with a view to providing insight into the design of more effective DHDPS inhibitors.

Functionally, DHDPS exists as a homo-oligomer (Fig 1B) [12]. Two DHDPS monomers self-associate to form a ‘tight-dimer’ (Fig 1B) [12], with the articulating surface between monomers capturing a significant proportion (10–13%) of the total subunit surface area [12]. Dimerization of DHDPS is crucial to enzyme function since monomeric DHDPS mutants demonstrate attenuated enzyme activity and decreased substrate binding affinity, particularly for pyruvate, which is the first substrate to bind DHDPS [13–15]. Each DHDPS monomer adopts a classical TIM-barrel fold that encloses a 30 Å-long cavity where the enzyme active site is encapsulated [16, 17]. Several key catalytic residues have been identified using X-ray crystallography and site-directed mutagenesis [16, 18, 19]. A catalytic triad of two tyrosines (Tyr109, Tyr135, *S. aureus* numbering) and a threonine (Thr46, *S. aureus* numbering) function as a proton relay during catalysis (Fig 1C) [20]. Dimerization of DHDPS allows for Tyr109 to interdigitate across the dimer interface, completing the catalytic triad of the adjacent monomeric unit and concomitantly creating two equivalent active sites per DHDPS dimer. Another residue, Lys163 (*S. aureus* numbering), forms a Schiff-base with pyruvate during catalysis [18]. These residues are virtually indispensable for enzyme function [20–22]. Crossing the lip of the active site cavity is the solvent-exposed residue Arg140 (*S. aureus* numbering), which has been implicated in the role of stabilizing the catalytic triad and presumably binding of substrates, particularly ASA [16, 23]. However the precise mechanism of Arg140’s role in DHDPS remains poorly understood.

Molecular dynamics (MD) simulation has emerged as a useful tool for gaining insight into various biological phenomena, such as enzyme allostery [24], protein dynamics [25], and binding of small molecules to their cognate protein receptors [26–28]. Multiple independent simulations can be performed that follow the unbiased motion of ligands in and around the binding site, allowing for identification of various factors that contribute to the interaction, such as residue bonding networks and protein conformational change [26]. Large ensembles of trajectories from such simulations can be pooled and clustered into microstates based on criteria such as root-mean square deviation (RMSD). The statistics of transitions between microstates across the trajectory ensemble can then be used to create a Markov state model (MSM) that captures the essential dynamics of the process [29, 30]. These models can capture the kinetics of binding events and allow for thermodynamic quantities to be calculated.

In this work, we present a detailed description of the binding dynamics of the substrate pyruvate and of the enzyme DHDPS. We use all-atom MD simulations to completely recapitulate the entire pyruvate binding process from bulk solvent to the crystallographic bound pose. By means of MSMs, we find that there are several key and metastable intermediates in this pathway defined as ‘hotspots’. Addressing the results from this study, the long-term goal of this project is to design DHDPS inhibitors that incorporate the targeting of this binding intermediate.

## Results

To date, approximately 80 structures of DHDPS have been deposited in the RCSB Protein Data Bank (www.rcsb.org/pdb/). Several of these structures have been co-crystallized with ligands including the first substrate to bind in the active site: pyruvate. However, comparison of the apo (i.e. unliganded) and pyruvate-bound structures provides only start and end points for understanding ligand binding, leaving a void of structural information defining the binding dynamics of pyruvate to the active site of DHDPS. To build a complete picture of substrate binding, a dynamic approach is required. Accordingly, we have employed all-atom MD simulation to describe the binding dynamics of DHDPS towards its first substrate, pyruvate, with a view to bridging this gap.

### Starting Structure

The choice of starting structure is an important decision in any MD simulation study. We employed the structure of DHDPS from the bacterium *S. aureus* (PDB ID 3DAQ) [12]. The rationale for selecting *S. aureus* DHDPS as the subject of this study was made on the basis of two considerations. Firstly, it adopts a non-canonical dimeric assembly that is in contrast to tetrameric orthologs from the majority of other plant and bacterial species [12, 31, 32]. This provides a considerable cost-saving in computational time (i.e. approximately two-fold reduction in the number of protein non-hydrogen atoms required). Secondly, DHDPS from *S. aureus* has been well-characterized structurally, providing both pyruvate-bound and apo structures as reference points [12, 33]. The availability of these structures provides both start and end configurations of DHDPS against which we can make direct comparisons with our own computational results.

### MD Simulations of DHDPS and Pyruvate

We investigated pyruvate binding to DHDPS using all-atom MD simulation. The process of ligand binding to an enzyme active site must inherently be dynamic, requiring small-scale (e.g. residue side chain motion) and potentially large-scale receptor motions (e.g. conformational dynamics). Sampling the entirety of these motions within a single extended trajectory is both impractical and computationally inefficient. Thus, to broaden our coverage of the conformational landscape of DHDPS we generated an ensemble of different DHDPS configurations by sampling from a 100 ns MD simulation. Each of these configurations were used as starting points for new rounds of minimization.

### Binding of Pyruvate

For each of our systems we simulated DHDPS with two molecules of the substrate pyruvate. We placed each of these ligands at locations distal to the crystallographic binding site (i.e. the DHDPS active site) using a randomized placement protocol (see Methods for details). This step was included to alleviate imposing a bias on the binding pathway. We first conducted pilot MD simulations of the system and observed evidence of pyruvate binding into the DHDPS active within 10–100 ns. Thus we extended the simulation data set by performing 80 MD simulations ranging between 10 ns and 100 ns for a total simulation time of 8.4 *μ*s per molecule of pyruvate, yielding a mean simulation length of 52.4 ns (± 30 ns).

**Fig 2:**
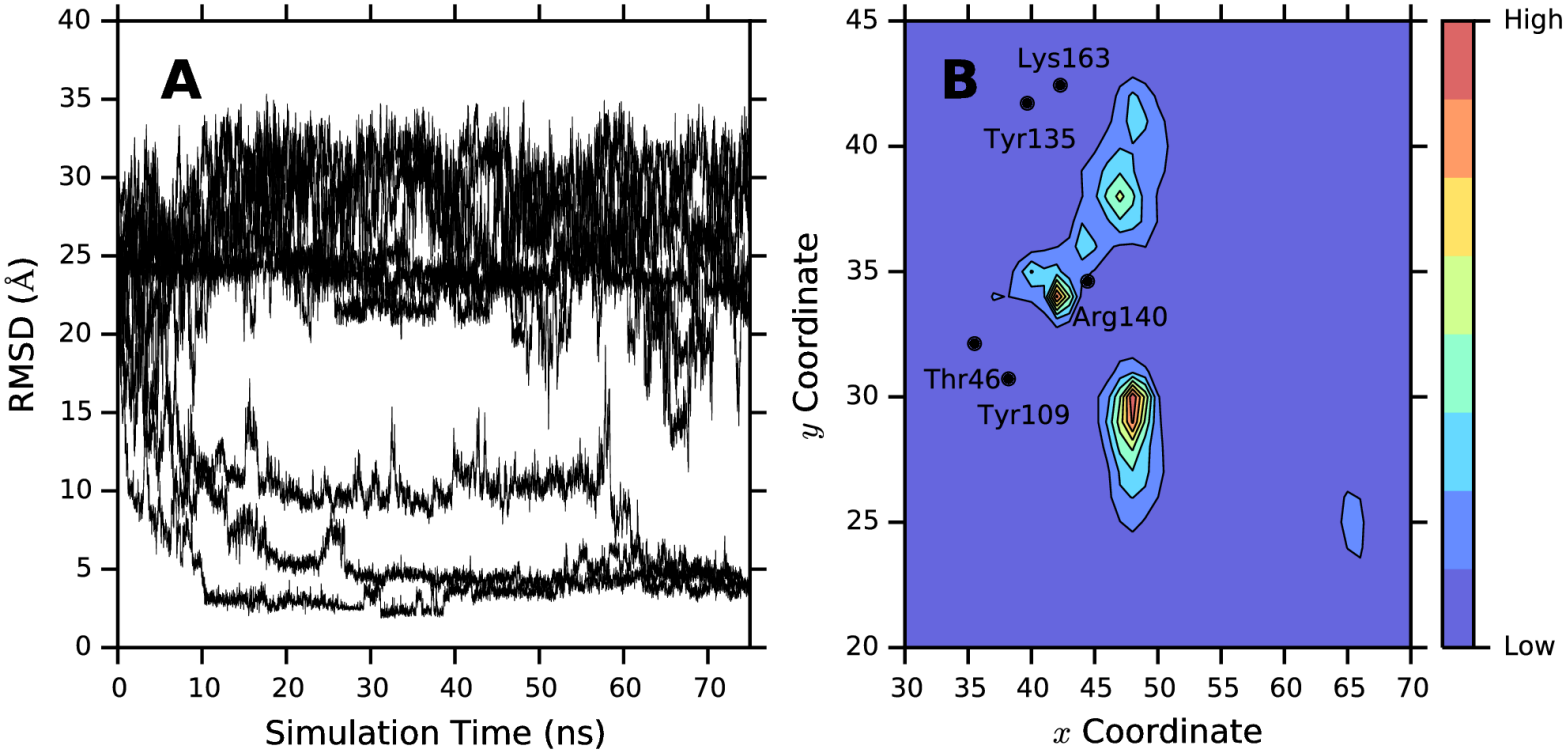
Binding data. (A) Ligand RMSD to crystal structure (PDB ID 3DI1) is shown as a function of time for 10 randomly selected trajectories, representing an eighth of the total simulation data set. Hydrogen atoms were excluded from RMSD calculations. (B) Ligand density plot. The *x* and *y* plane components of the geometric center of pyruvate were derived from each frame of the simulation data set and binned to form a 2-dimensional matrix. The color mapping reflects the number of counts within each of these bins (blue indicates low density, red indicates high density). For reference, the relative locations of several active site residues (Thr46, Tyr109, Tyr135, Arg140, and Lys163; *α*-carbons only) are indicated using black markers and labelled accordingly.

Many of our simulations capture ligand binding events. We track binding by calculating the root-mean square deviation (RMSD) of pyruvate heavy atoms (i.e non-hydrogen atoms) against a reference structure of pyruvate in complex with DHDPS (PDB ID 3DI1) [33], correcting first for translational and rotational protein motions (Fig 2A). In 5 of our simulations the RMSD converges upon a value of less than 2.3 Å(Fig 2A). In some simulations this is achieved after as little as 12 ns of simulation time. Transitions from this converged position are rare (i.e. the vast majority of simulations remain in this state for the remainder of the simulation). Some simulations show remarkable agreement with the reference structure, achieving conformations that deviate by as little as 1.85 Å. These findings suggest that our simulations are able to recover the crystallographic ‘bound pose’ with reasonable resolution in the absence of *a priori* knowledge of the binding trajectory.

Furthermore, our MD simulations identify several pyruvate interaction ‘hotspots’ (assessed by spatial residence of pyruvate; Fig 2B). Mapping out the regions of high pyruvate density (‘hotspots’) relative to DHDPS, we find that many of these appear spatially proximal to key active site residues such as Thr46, Tyr109, Tyr135, Arg140, and Lys163 (Fig 2B). Similar clustering patterns are observed when comparing other 2-dimensional planes (S1 Fig). For reference, we provide an example of a single, 75 ns-long binding trajectory in S1 Video. The majority of simulations, however, do not reach the bound pose even up to simulation time-scales of 75 ns (Fig 2A). Furthermore, no complete dissociation events to solvent from the bound pose occur. Nonetheless, the simulation data available is sufficient for a comprehensive analysis of binding kinetics.

### Markov State Model

We submitted our MD simulation data to more exhaustive analysis of pyruvate binding kinetics by building a MSM. The first step in this process involved assigning structurally-related pyruvate conformations into clusters. In brief, we found that partitioning our simulation data set (over 5.5 million conformations) into 738 clusters provided sufficient resolution for the resulting model, with the mean cluster radius calculated as 2.6 ± 0.4 Å. By measuring the time-dependence of relaxation time-scales in our model (S2 Fig), we found that the Markov property was satisfied using lag times at and beyond 3 ns. Thus, we built an initial fine-grained MSM using a lag time of 4 ns. S3 Fig shows that the states comprising this fine-grained model are densely interconnected.

**Fig 3:**
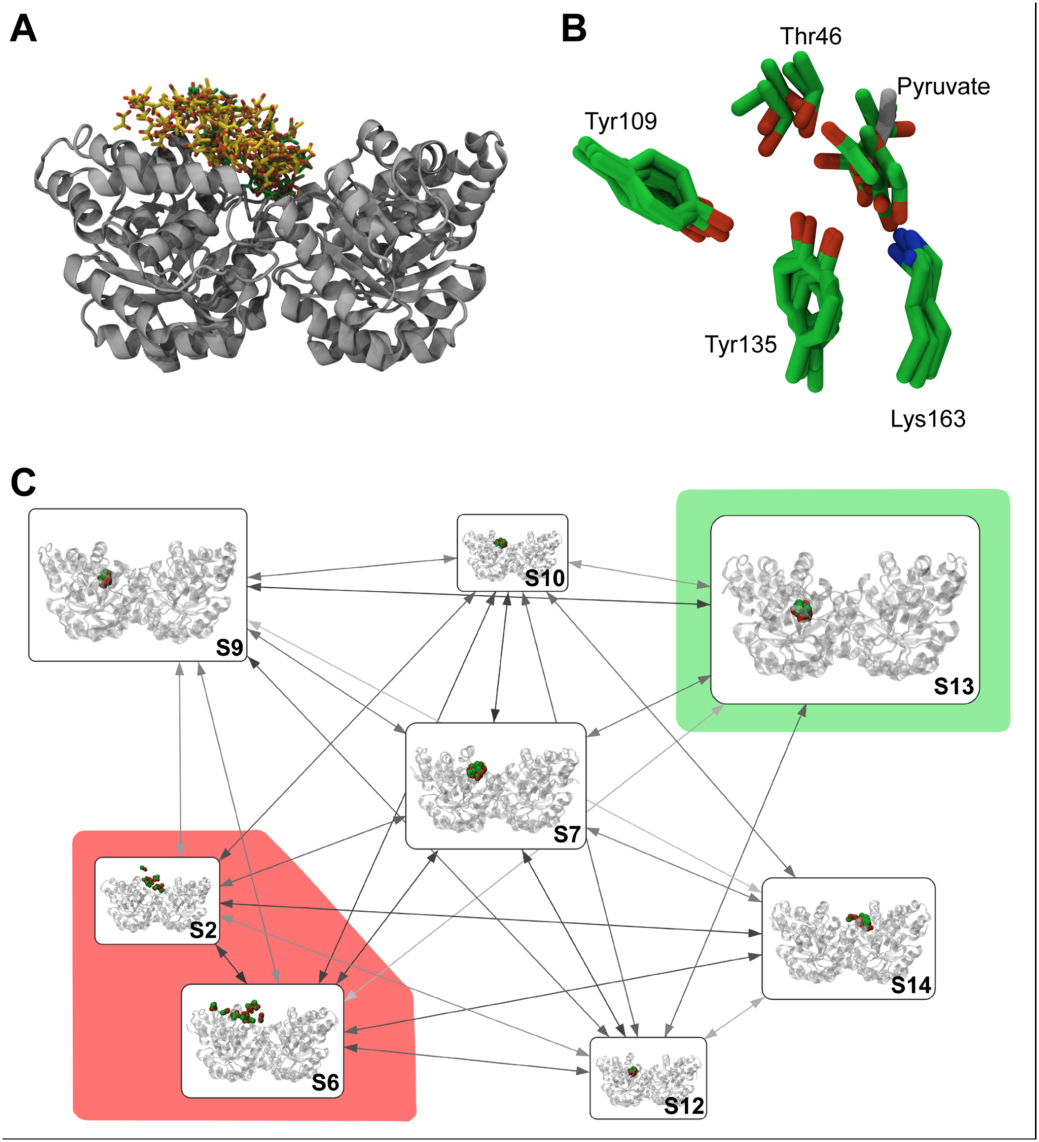
Grouping of states from binding trajectories into a coarse-grained metastable state model. (A) Unbound states. One hundred randomly selected conformations of pyruvate from unbound states S2 (yellow) and S6 (green) are presented against a cartoon representation of DHDPS. Collectively, poses within these states lack any conserved interactions with the protein surface. (B) The DHDPS-pyruvate bound complex (S13). Conformations of pyruvate and active site residues from this state (green) are contrasted with a crystallographic reference structure of pyruvate bound to DHDPS (PDB ID 3DI1; silver). Key active site residues Thr46, Tyr109, Tyr135, and Lys163 are indicated. Conformations of pyruvate within this state deviate from the reference structure by as little as 1.85 Å. Note that Tyr109 is shown from the opposing DHDPS subunit. (C) Individual metastable states, labelled accordingly, are shown as nodes within rounded boxes. Edges depict bidirectional interstate transitions, where edge shade reflects the transition probability (darker arrows indicate higher probabilities). States classified as unbound (S2, S6) are shaded in red, whereas the DHDPS-pyruvate bound state (S13) is shaded in green. For clarity, only highly-populated states (equilibrium populations greater than 4%) are shown.

Such fine-grained models, while a resource of quantitative information, suffer from a high degree of complexity that makes analysis difficult. With this in mind, and with a view to providing a more readily-interpretable representation of our model, we grouped states from our fine-grained model into a series of long-lived and metastable states (S) (Fig 3). We used a Bayesian approach [34] to assign kinetically-related states into a metastable MSM (Fig 3). The resulting metastable MSM identifies 17 states (S0–S16), each describing components of the pyruvate binding pathway. In Fig 3C we make use of a network diagram to show the most highly populated of these states (i.e. those with equilibrium probabilities in excess of 4%). These states correspond to S2, S6, S7, S9, S10, S12, S13, and S14. Here, representatives from each macrostate are depicted structurally as nodes with interstate transition probabilities indicated by the shade of connecting arrows (darker arrows indicate greater probability).

The starting point for pyruvate binding in the coarse-grained model is depicted in Fig 3A and 3C as two states: S2 and S6. These represent unbound states of pyruvate, given that ligand conformations within S2 and S6 are distal to the active site and lack any substantial points of interaction with DHDPS residues. We next evaluated the relevance of other metastable states in our model (S0–S1, S3–S5, and S7–S17) to pyruvate binding by making comparisons with available experimental data, using structural similarity to the pyruvate-bound DHDPS crystal structure (PDB ID 3DI1). For this we used two measures: (i) the average RMSD of pyruvate heavy atoms relative to key active site residues, and (ii) intermolecular bonding patterns between protein and ligand. Under these conditions we find that S13 best captures the bound pose (Fig 3B), since this state demonstrates the best agreement with the pyruvate-bound enzyme structure (RMSD 3.69 ± 0.72 Å) (Fig 3B). Moreover, S13 includes the formation of a hydrogen bond network with key active site residues, namely Thr46, Tyr109, and Tyr135, in a remarkably similar geometry to the X-ray structure [33]. This is exemplified in Fig 3B.

### Pyruvate Binding Pathways

From our metastable MSM we made use of transition path theory (TPT) [35, 36] to investigate pathways of pyruvate binding. The output of such analyses is a collection of paths that describe the routes by which the system can unidirectionally traverse from unbound states, S2 and S6 (Fig 3A and 3C), to the bound state S13 (Fig 3B and 3C), which are then ranked according to reactive flux. In S4 Fig we show that the top six pathways capture almost three quarters of the total binding flux.

**Fig 4:**
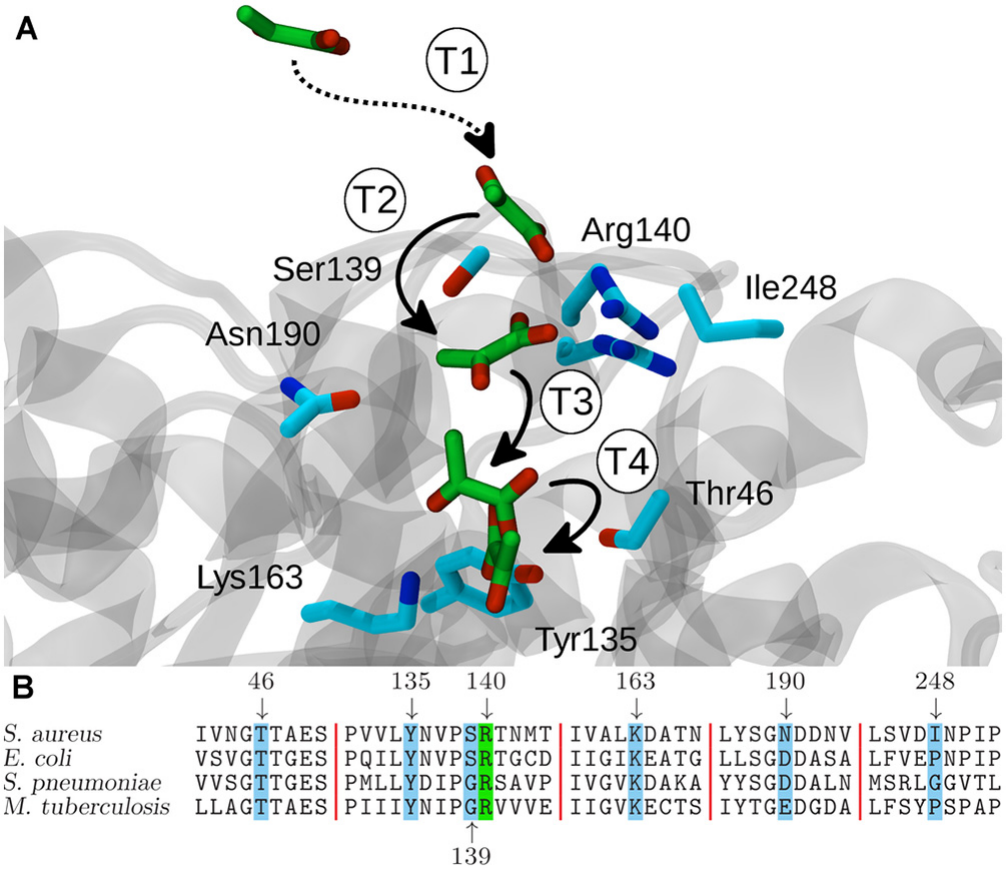
The major pyruvate-binding pathway is multi-step. (A) Pyruvate, indicated using green carbon atoms, must successively pass through several binding intermediates to reach the crystallographic bound pose. From bulk solvent, pyruvate forms a transient interaction with an arginine residue at the entryway to the active site (T1), moves into the active site cavity (T2), and in the penultimate step penetrates deeper into the active site to assume a ‘pre-bound’ pose (T3). Finally, from the ‘pre-bound’ pose pyruvate undergoes a twisting motion (T4) and achieves the crystallographic DHDPS-pyruvate complex. (B) Multiple sequence alignment of bacterial DHDPS enzymes. Several interacting residues from the binding pathway depicted in (A) are absolutely conserved across species. Sequence alignment was performed using CLUSTALO [37].

We first examine the dominant binding pathway, comprising four transitions. We label each of these transitions sequentially from T1 to T4 (Fig 4A). In the first transition (T1) pyruvate partitions from bulk solvent (S6) to an interacting surface of DHDPS (S7). Here, pyruvate is stabilized by the establishment of a salt bridge between the carboxylate moiety of pyruvate and the guanidinium group of the solvent-exposed Arg140 residue of DHDPS. In the second transition (T2), the side chain of the interacting Arg140 demonstrates a high degree of flexibility (Fig 4A). Specifically, Arg140 flips away from the bulk solvent towards the active site cavity, carrying pyruvate deeper into the entryway of the active site (S12). The carboxylate-guanidinium salt bridge between pyruvate and Arg140 is sacrificed in the third transition step (T3) as pyruvate migrates further into the active site cavity (Fig 4A). The loss of this interaction with Arg140 in the penultimate step is balanced by the establishment of a new hydrogen bond network with several active site residues such as Thr46, Tyr135, and Lys163, allowing the substrate to enter into a ‘pre-bound’ pose (S9). It is from this ‘pre-bound’ pose that pyruvate undergoes a twisting and flipping motion (T4) to assume the pyruvate-DHDPS complex, or bound pose (S13)[33].

Alternative pathways show noteworthy deviations from the dominant pathway shown in Fig 4A. Many of these alternative paths allow for pyruvate to transition to the bound state whilst boycotting the ‘pre-bound’ intermediate S9, suggesting that this may not be an essential requirement for pyruvate binding. Likewise some of the highest flux pathways boycott the binding intermediate S12. Other paths allow for greater flexibility at the entryway to the active site, instead relying on transient non-covalent interactions with other residues proximal to the active site (e.g. Ile248). Compellingly however, the six highest flux pathways have a strict requirement for the formation of the pyruvate-Arg140 salt bridge intermediate (S7), suggesting the S7-intermediate is a highly-favorable event towards pyruvate binding. Consistent with these findings, an *Escherichia coli* DHDPS mutant incorporating alanine at the equivalent position of Arg140 demonstrates ~1000-fold reduced enzyme activity and decreased affinity for substrate [23]. This may be expected, given the absolute conservation of Arg140, and other key catalytic residues (Thr46, Tyr109, Tyr135, Lys163) in bacterial DHDPS sequences (Fig 4B).

### Umbrella Sampling

**Fig 5:**
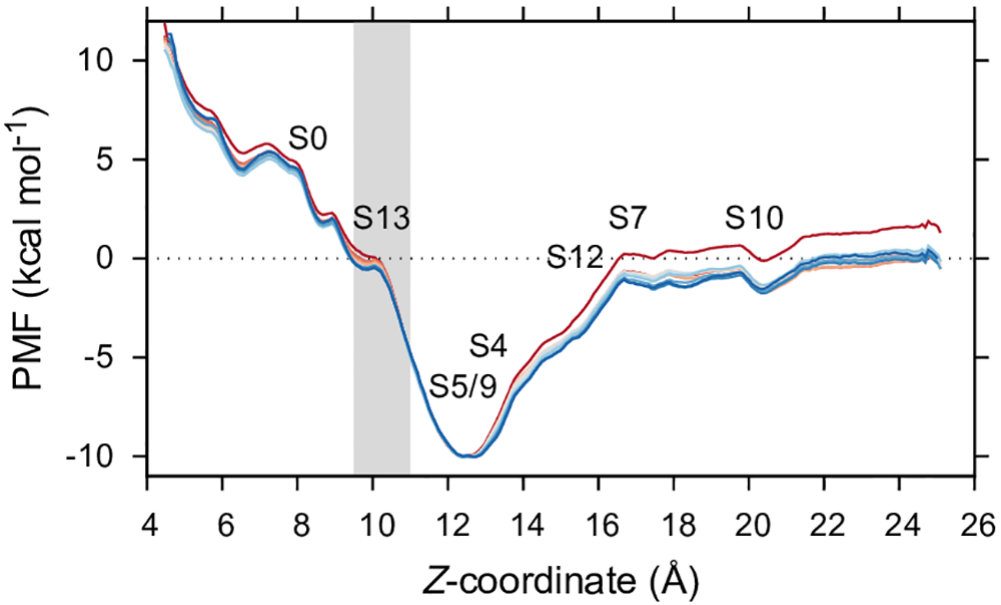
Umbrella sampling PMF. PMF curve calculated using 28 windows of umbrella sampling along an arbitrary *Z*-coordinate. Each curve, colored from red to blue, represents successive truncation of the data in 5% increments from the beginning of each simulation window until the final 50% of data (2.5 ns) remained. Several bound states identified are labelled according to their average *Z*-coordinate. State S13, which was bimodal with respect to the *Z*-coordinate, is highlighted as a gray box.

Umbrella sampling was used to determine the relative free energies of metastable binding states using a simple one-dimensional coordinate along the axis of the pyruvate binding pathway (depicted in S1 Fig). This coordinate was mostly sampled in windows of 1 Å, while windows centered on 8, 9, 14 and 19 Å experienced unacceptably long de-correlation times (≈1 ns). Thus, these windows required a finer sampling of 0.5 Å with a greater biasing force to reduce this the de-correlation time to no greater than 200 ps. Fig 5 shows that 5 ns of simulation time for each window was sufficient to converge a potential of mean force (PMF) curve along this coordinate. Error estimates, de-correlation times, and force constants for each window are included in S1 Table for reference, and histograms shown in S6 Fig. The PMF profile in Fig 5 shows that the local energy minimum corresponding to S10 has an associated free energy difference of (-0.5 ± 0.2 kcal mol^-1^), while a similar energy minimum is observed for the *Z*-coordinate equivalent to S7 (-0.2 ± 0.2 kcal mol^-1^) which, as Fig 4 shows, is en route to the binding intermediate S12 (-3.0 ± 0.2 kcal mol^-1^). S12 presents as a relatively higher-energy intermediate between states S4 (-8.6 ± 0.2 kcal mol^-1^) and S5/S9 (-9 ± 0.2 kcal mol^-1^), the latter of which forms the global minimum of the PMF profile. Surprisingly, the bound state S13 (-3.0 ± 0.2 kcal mol^-1^), which was bimodal with respect to the *Z*-coordinate (Fig 5, gray box), appears as an energetic intermediate between S5/S9 and the least energetically favorable state along the PMF profile, S0 (0.5 ± 0.2 kcal mol^-1^). These umbrella sampling data support that the observed states in the model show in Fig 4 are indeed thermodynamically metastable.

### Gross Motions Are Not Induced By Pyruvate Binding

Large-scale changes to protein structure and dynamics upon ligand binding are not uncommon [38, 39]. Thus, we considered whether similar large-scale dynamic motions were at play in the case of DHDPS pre- and post-binding pyruvate. Comparison of apo and pyruvate-bound crystal structures of several bacterial DHDPS enzymes reveals only minor structural differences (.12–.19 Å; S2 Table). Thus, it was not anticipated that DHDPS would undergo substantial structural changes after transitioning from the apo to pyruvate-bound form. Indeed, there remained no substantial perturbation of DHDPS conformation (S7A Fig) or residue fluctuations (S7B Fig) up to 60 ns after transitioning into the bound form (S13). Previous MD simulations performed using the crystal structure of DHDPS (PDB ID 3DAQ) over longer time-scales indicate that while the enzyme is afforded a degree of inter-subunit flexibility (so called “enzyme-breathing”), intra-subunit motions remain minimal [40]. Thus, our results are consistent with these earlier reports. We reason that these data provide compelling evidence that macroscopic, intra-subunit protein motions play a limited role in pyruvate binding. It remains to be seen whether this is the case for subsequent catalytic steps to product formation, such as binding of the second DHDPS substrate, ASA (Fig 1A).

## Discussion

DHDPS is a promising antibacterial drug target. However, despite decades of conventional rational drug design and the determination of more than 80 DHDPS structures, potent inhibitors remain elusive [3, 10, 11]. We suggest that a major shortcoming of previous studies is a lack of dynamic information describing ligand binding to druggable sites of DHDPS. Thus, the primary goal of this study was to identify and characterize the dynamics of ligand binding to the potentially druggable active site of DHDPS. More specifically, we set out to map the binding pathway for the DHDPS substrate pyruvate in atomistic detail using MD simulation. Importantly, our simulations recover the bound pose observed in the crystallographic structure with remarkable precision, allowing for robust conclusions to be articulated concerning the binding trajectory. We find that (i) Arg140 side chain motion correlates with the recruitment of pyruvate from solvent in a role we define here as a ‘gateway’ residue; (ii) the majority of binding passes through a transient state involving a salt bridge between the carboxylate and guanidinium moieties of pyruvate and Arg140, respectively; (iii) pyruvate binding, independent of catalysis, is a dynamic phenomenon with several distinct metastable states described here as ‘hotspots’; and (iv) pyruvate binding is an energetically favorable event with discrete thermodynamic intermediates.

Firstly, with regards to Arg140’s role as a ‘gateway’ residue, it is interesting to compare our *in silico* findings with previous structural [16] and kinetic [23] reports *in vitro*. Structural studies by Mirwaldt et al. [16] put forward a potential role for Arg140 in substrate recruitment given the positioning this residue at the ‘gateway’ to the active site. Whereas mutation of the Arg140 equivalent in *E. coli* DHDPS was shown to markedly reduce catalytic activity (i.e. decreased *k*_cat_) and increase the apparent Michaelis constant (*K*_M_) for ASA by ~50-fold, only a subtle increase in the apparent *K*_M_ was found for pyruvate [23]. However, what remains to be explained is the molecular mechanism that underpins the ~50-fold increase in the apparent *K*_M_ for ASA. We propose a similar, but more pronounced, ‘gateway’ role for Arg140 during the subsequent step of the DHDPS-catalyzed reaction, when ASA is recruited (Fig 1). It would thus be of interest in future studies to explore the binding dynamics of DHDPS with ASA using similar *in silico* approaches to those reported here for DHDPS-pyruvate interactions.

Secondly, to validate our second conclusion regarding the crucial transition intermediate involving the carboxylate of pyruvate and the guanidinium of Arg140, future studies could explore the importance of this phenomenon by substituting the side chain and/or ligand moieties with uncharged equivalents. For example, MD simulations could be performed on a mutant *S. aureus* DHDPS structure incorporating alanine at position 140, which would replace the positively-charged guanidinium moiety with a shorter-chain methyl group. Indeed, this mutation has already been explored kinetically *in vitro*[23]. Alternatively, the carboxylate group of pyruvate could be replaced by an electrostatically neutral aldehyde group. Either of these chemical transmutations would provide further insight into the chemical requirements of this transition.

Thirdly, we recover similar binding energies for pyruvate in our *in silico* model compared to those experimentally determined *in vitro*. Moreover, we calculate that the relative energy difference between the apo- and pyruvate-bound state correlates to approximately -10 kcal mol^-1^ (Fig 5). This agrees well with isothermal titration calorimetry measurements [22]. Importantly, these data imply that intermediate states observed in our model are indeed thermodynamically metastable and are not likely attributable to short-lived artifacts.

Defined thermodynamically-metastable ligand binding/unbinding intermediates appear to be broadly applicable to many protein-small molecule interactions [26–28, 41]. For example, in a thematically related computational study Da et al [41] indicated that egress of inorganic pyrophosphate from the active site of yeast RNA polymerase II adheres to a four-state model. In this model Da et al [41] showed that transitions between these kinetically metastable ‘hotspots’ is principally mediated by mutable electrostatic interactions between protein lysine or histidine residues and the ligand pyrophosphate. Furthermore, *in silico* mutation of these residues was found to retard product release from the RNA polymerase II active site [41]. Relating this back to our current study, it would be a worthwhile endeavour for future studies to measure whether transmuting residues involved in the pyruvate binding transition states, such as Thr46, Tyr109, Tyr135, Arg140, Lys163, or Ile248, alter the kinetics of substrate entry to the DHDPS active site.

In conclusion, this study sheds light on the binding pathways of an important enzyme-substrate interaction that has identified several metastable binding intermediates with distinct protein-ligand interaction profiles (i.e. ‘hotspots’). We suggest that rational drug design can be augmented for next-generation DHDPS-inhibitors by considering the outcomes of this study.

## Methods

### Ligand Preparation

The structure of pyruvate was drawn and optimized using Avogadro 1.1.0 [42]. Topology and parameter files for pyruvate were generated with SwissParam [43]. The protonation state was determined using Marvin Sketch 5.11.5 (ChemAxon, Budapest, Hungary; http://www.chemaxon.com/), appropriate to a solution pH of 7.0.

### Model Preparation

The high-resolution (1.45 Å) crystal structure of DHDPS from the bacterium *S. aureus* (PDB ID 3DAQ) [12] was utilized for MD simulations. Protein chains C and D, both artefacts of crystal packing, were removed. Crystallographic waters were discarded and missing hydrogens added using VMD [44]. The structure was solvated in a rhombic dodecahedron using TIP3P waters [45] extending at least 12 Å from any protein atom. Na^+^ and Cl^-^ were added at a set concentration of 150 mM, with additional Na^+^ added to neutralize the total charge of the system. This process required a total of 18,975 waters, 80 Na^+^, and 54 Cl^-^ ions.

### Simulation Parameters

Molecular dynamics data were generated with NAMD 2.9 [46], using the CHARMM22 force field with CMAP corrections [47, 48]. Temperature was maintained at 310 K using a Langevin thermostat (damping coefficient 5 ps^-1^), and system pressure was adjusted to 1 atm by use of a Langevin piston barostat. Periodic boundary conditions were implemented. Long range electrostatics were computed using the Particle Mesh Ewald Method [49], applying a nonbonded distance cut-off of 12 Å. Covalent bonds associated with hydrogen atoms were restrained using the SHAKE algorithm [50], allowing for an integration time-step of 2 fs. Trajectory frames were recorded at simulation time intervals of 10 ps. VMD 1.9.2 [44] was used for trajectory analysis unless otherwise specified.

### Minimization and Equilibration

Systems were first minimized for 5000 steps of steepest descent followed immediately by 8 ns of simulation during which the global protein backbone RMSD was allowed to equilibrate, priming the system for production runs. The final frame of this equilibration run was used as a starting reference for a longer 100 ns production run.

### Ligand Binding Simulations

80 replicas of the DHDPS system were seeded by sampling from the 100 ns production run at evenly spaced time intervals. This provided an ensemble of different starting protein configurations. Two ligand molecules were randomly placed in each of these systems using Packmol [51], imposing a distance limit of 6 Å from any protein atom and at least 15 Å from the *ζ*-nitrogen atom of Lys163 of protein chain B. Overlapping waters were removed. A restraint was placed on the carbon atom of the ligand carboxylate group such that the upper limit of searchable space in the system was restricted to a radius of less than 26 Å from Lys163-N*ζ*. A schematic of the simulation set-up is given in S8 Fig. Simulations were run for between 10 ns to 100 ns as computing resources allowed.

### Clustering

553,200 conformations were obtained from the simulation data set. These conformations were clustered using the hybrid k-centers k-medoids algorithm implemented within MSMBuilder2 2.7 [52], using a reduced selection of the total simulation data set sub-sampled at intervals of 250 ps. Clustering was performed based upon the RMSD of pyruvate heavy atoms to produce a fine-grained, 738-state model. Protein backbone alignment using both protein chains was carried out prior to clustering. Clustering was performed using MSMBuilder2 2.7 [52]. Following clustering, the remaining conformations from the simulation data set were assigned to these clusters. The average cluster radius in the fine-grained model (± standard deviation) was found to be 2.6 ± 0.4 Å, indicating that clusters are sufficiently tight to provide a high degree of resolution.

### Markov State Models

MSMs in this study were constructed based upon methods described elsewhere [26], using a lag time of 4 ns (see S1 Text for details), allowing us to construct a highly detailed microstate representation of the binding pathway. Such microstate representations are useful for examining kinetic properties of MSMs, but are often too complex to be readily understandable. To address this shortcoming we coarse-grained the microstate model into a simpler, 17-state model. The number of states was selected by examination of the Bayes factor using similar principles reported by [53]. Observations made from coarse-grained MSMs are useful in that they can allow for comparison with experiment. For this study, we used a Bayesian method [34] as implemented in MSMBuilder2 2.7 [52] to perform coarse-graining.

### Transition Path Theory

The major flux pathways connecting the unbound state to the bound state were investigated using TPT [35, 36] as implemented within MSMBuilder2 2.7 [52]. In our calculations, we considered S2 and S6 as the unbound states, and S13 as the bound state.

### Umbrella Sampling

PMF profiles were obtained using umbrella sampling methods with the collective variable module within NAMD [46]. A one-dimensional binding coordinate (*Z*-coordinate) was defined using two arbitrary reference points designed to create an axis that encompassed the binding pathway depicted in Fig 4. The binding, or *Z*-coordinate, was sampled in windows of 0.5 Å or 1 Å, using harmonic force constraints of 10.0 kcal mol^-1^ and 7.5 kcal mol^-1^, respectively. The geometric center of non-hydrogen pyruvate atoms was restricted to within a radius of 7.5 Å by a boundary force of 10.0 kcal mol^-1^ from the *Z*-axis. This was imposed to reduce the degrees of freedom. Additionally, the C*α* of the protein were constrained to within 0.5 Å of their starting positions by a 10.0 kcal mol^-1^ force constraint to prevent rotational and translation drifts that may affect the definition of the *Z*-coordinate.

PMF curves were calculated using the weighted histogram analysis method (WHAM) [54]). *Z*-coordinate measurements were captured every 0.2 ps. Convergence of the dataset was assessed by segmentally truncating the simulation data within each window to reduce the sample size. Reported free energies were calculated from the final 2.5 ns of simulation time and errors attributed using 100 Monte Carlo bootstraps with time correlation values equal to the time taken for an autocorrelation to decay to 1/*e*.

## Supporting Information

S1 Video Single simulation of pyruvate binding to DHDPS. Side-chains of several active site residues (Thr46, Tyr107, Tyr135, Arg140, and Lys163) are shown using stick representations. The video plays at a constant 0.04 ns frame^-1^, applying a smoothing window over 0.064 ns to both protein and ligand atoms. Water and ions have been omitted for clarity.
(M4V)

S1 Fig Ligand density plot. *x* and *z* (A) or *y* and *z* (B) components of the geometric center of pyruvate were derived from each frame of the simulation data set and binned to form a 2-dimensional matrix as described for Fig 2B. The color mapping reflects the number of counts within each of these bins (blue indicates low density, red indicates high density). For reference, the relative locations of several active site residues (Thr46, Tyr109, Tyr135, Arg140, and Lys163; *α*-carbons only) are indicated using black markers and labelled accordingly. Comparison of the *x* and *y* components is shown in Fig 2B.
(TIF)

S2 Fig Lag time dependence. Plots for the top 10 slowest time-scales for the microstate model are shown. Implied time-scales flatten beyond approximately 3 ns. Error bars were assigned by performing five rounds of bootstrapping with replacement from the total trajectory data set (*N* = 160).
(TIF)

S3 Fig Microstate network model. Individual states are drawn as nodes of size proportional to their respective populations at equilibrium, and transitions between states shown as edges colored according to their respective transition probabilities using a red-white-black color scale (lower to higher probabilities, respectively). Node colors reflect coarse-grained state assignments.
(TIF)

S4 Fig Major flux pathways in the coarse-grained Markov state model (MSM). Individual pathways, indexed by reactive flux, are plotted as a function of their respective flux as a fraction of the total flux. The six highest-flux pathways (broken line) contribute approximately 66% of the total flux.
(TIF)

S5 Fig Umbrella sampling scheme. Graphical depiction of the umbrella sampling scheme. A representative pyruvate molecule is shown using a licorice representation for each of the 28 windows sampled (sequentially colored from red to blue) sampled along the *Z*-coordinate (black arrow). The *Z*-axis was defined using two arbitrary reference points indicated graphically as black spheres. A boundary condition restricting pyruvate to within 7.5 Å of the aforementioned axis is represented as a yellow cylinder.
(TIF)

S6 Fig Umbrella sampling bin density. Histograms of *Z*-coordinate measured during each window of umbrella sampling for PMF calculations. Data for each window was truncated from the beginning of the simulation in 5% increments until the final 50% of data remained (sequentially colored from red to blue).
(TIF)

S7 Fig Effect of pyruvate binding on DHDPS motions. (A) Overlay of protein backbone atoms for the first 10 ns (red) and final 10 ns (blue) of a pyruvate binding simulation (100 ns). Pyruvate achieved an RMSD to the crystal structure (PDB ID 3DI1) [33] of <2.3 Å after approximately 16 ns, remaining bound until the end of the simulation. Snapshots were taken at 1 ns intervals. For reference, the complete simulation is provided in S1 Video. (B) root-mean square fluctuation (RMSF) analysis of DHDPS residues over the first 10 ns (pre-bound; dark gray) and the final 10 ns (bound; light grey) from several pyruvate binding simulations (mean ± standard error of the mean (SEM), *n* = 4). Values were calculated only for protein chain B.
(TIF)

S8 Fig Enhanced sampling set-up. An upper limit restraint (26 Å) was placed upon the carboxylate carbon atom of pyruvate to restrict the searchable space available to the ligand. This restraint was relative to the position of the *ζ*-nitrogen of Lys163 from a single protein monomer with which pyruvate forms a Schiff-base during catalysis.
(TIF)

S1 Table Summary of WHAM of umbrella sampling windows. Each window was simulated a total of 5 ns (25050 points). Only the final 2.5 ns were used for WHAM analyses and autocorrelation.
(PDF)

S2 Table RMSD between apo and pyruvate-bound forms of DHDPS. (PDF)

S1 Text Supplementary material. (PDF)
