# Supplementary figures and images for: Dynamic Modelling Reveals ‘Hotspots’ on the Pathway to Enzyme-Substrate Complex Formation
Source: PLoS Comput Biol. 2016 Mar 11;12(3):e1004811. doi: 10.1371/journal.pcbi.1004811 (PMC4788353; doi:10.1371/journal.pcbi.1004811)

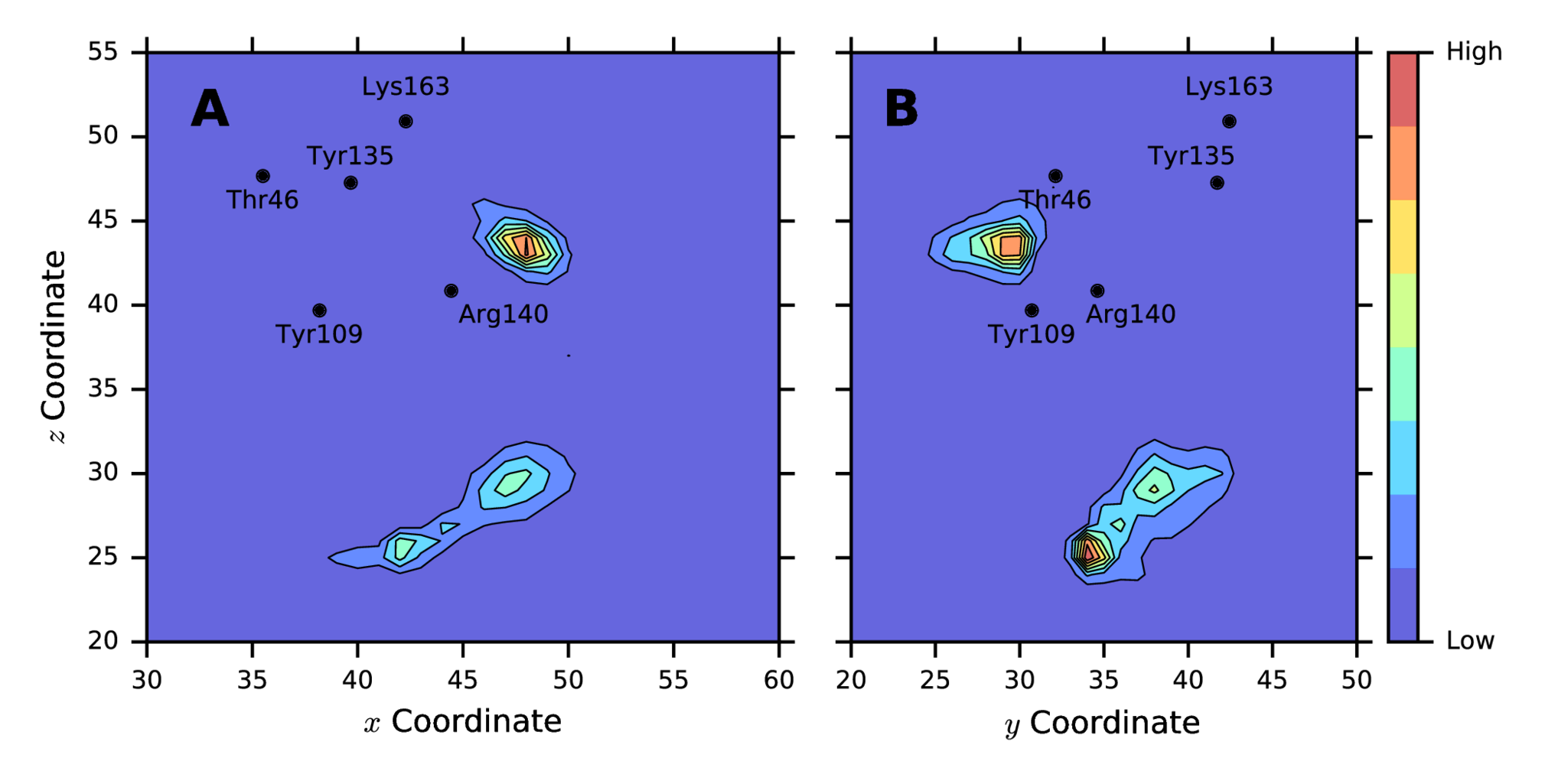

Supplement: S1 Fig — x and z (A) or y and z (B) components of the geometric center of pyruvate were derived from each frame of the simulation data set and binned to form a 2-dimensional matrix as described for Fig 2B. The color mapping reflects the number of counts within each of these bins (blue indicates low density, red indicates high density). For reference, the relative locations of several active site residues (Thr46, Tyr109, Tyr135, Arg140, and Lys163; α-carbons only) are indicated using black markers and labelled accordingly. Comparison of the x and y components is shown in Fig 2B. (TIF) [file pcbi.1004811.s002.tif]

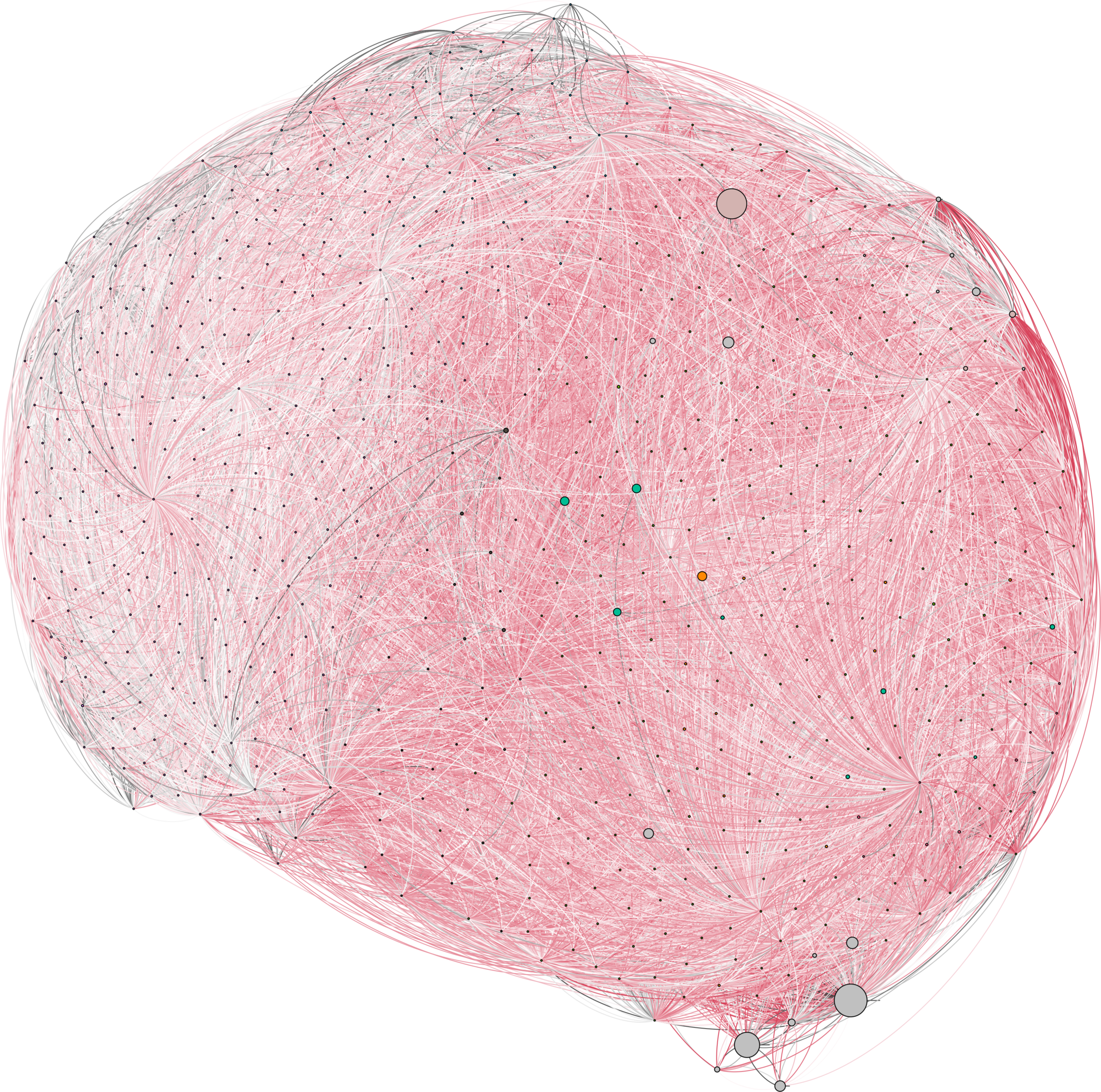

Supplement: S3 Fig — Individual states are drawn as nodes of size proportional to their respective populations at equilibrium, and transitions between states shown as edges colored according to their respective transition probabilities using a red-white-black color scale (lower to higher probabilities, respectively). Node colors reflect coarse-grained state assignments. (TIF) [file pcbi.1004811.s004.tif]

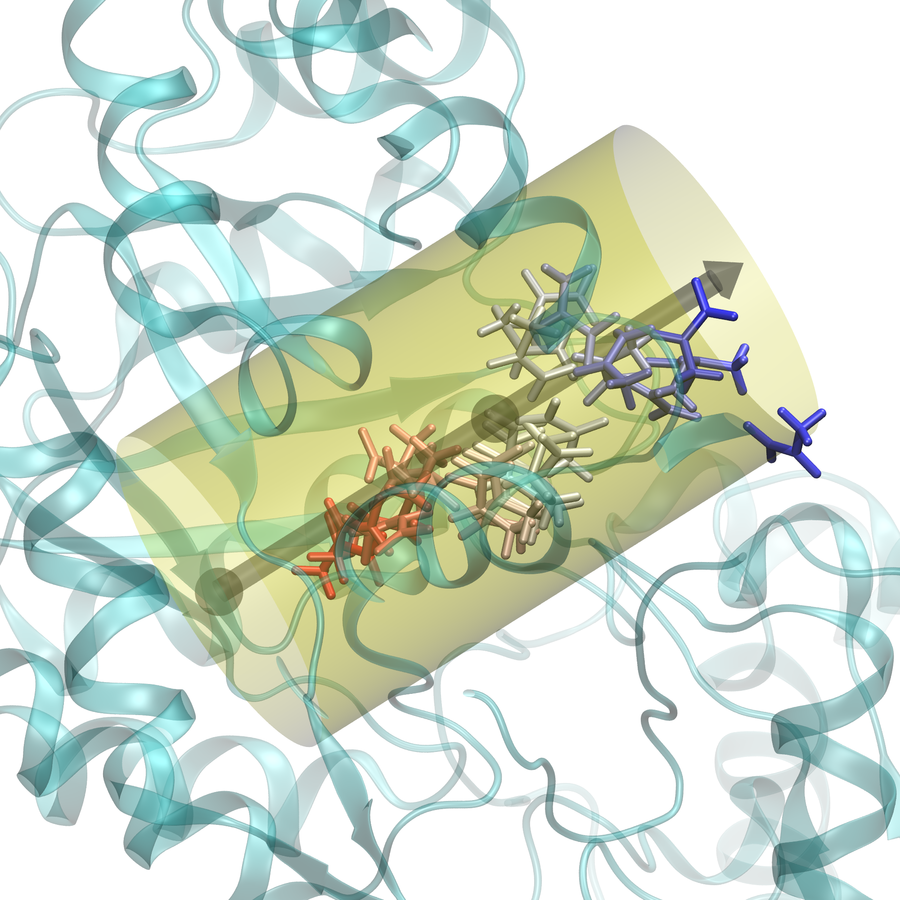

Supplement: S5 Fig — Graphical depiction of the umbrella sampling scheme. A representative pyruvate molecule is shown using a licorice representation for each of the 28 windows sampled (sequentially colored from red to blue) sampled along the Z-coordinate (black arrow). The Z-axis was defined using two arbitrary reference points indicated graphically as black spheres. A boundary condition restricting pyruvate to within 7.5 Å of the aforementioned axis is represented as a yellow cylinder. (TIF) [file pcbi.1004811.s006.tif]

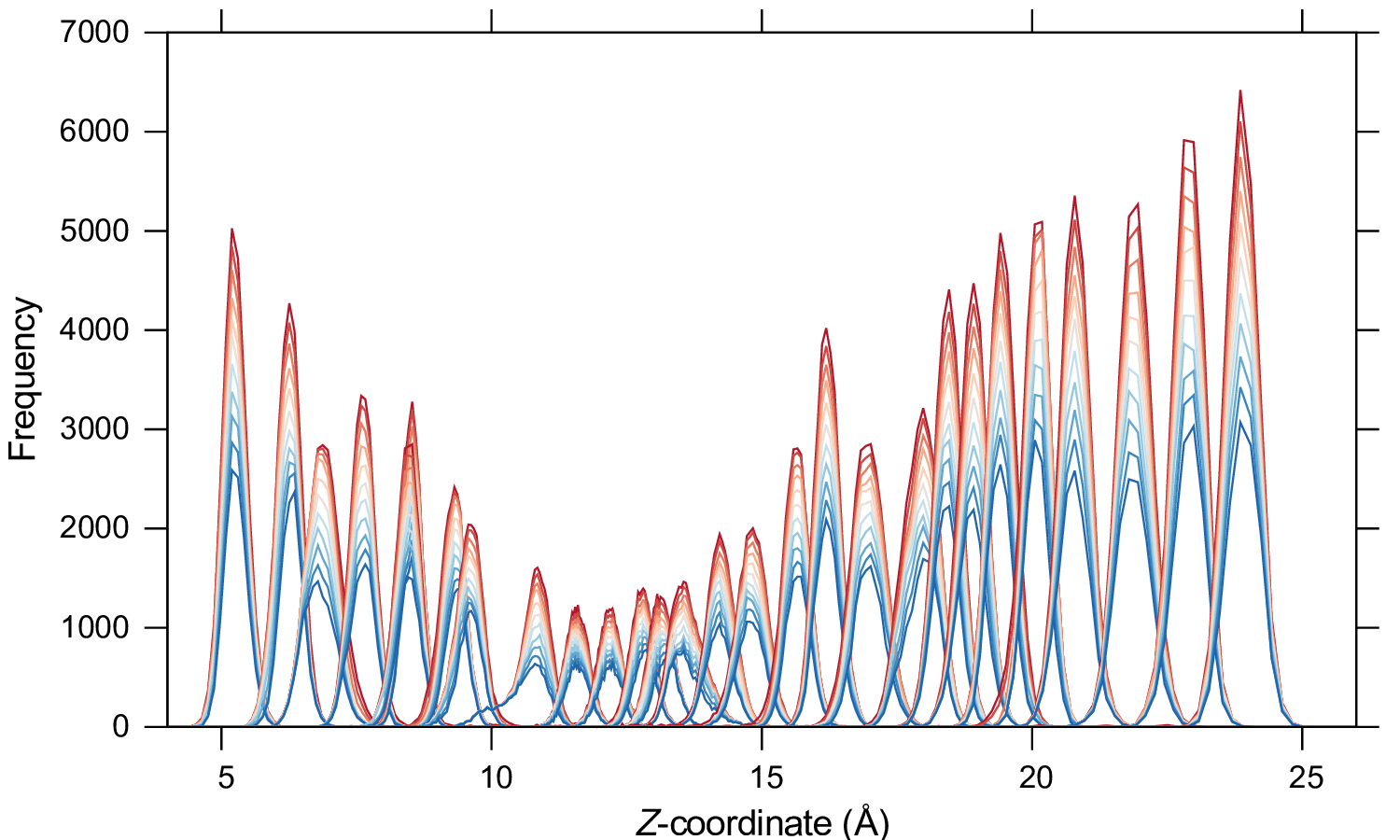

Supplement: S6 Fig — Histograms of Z-coordinate measured during each window of umbrella sampling for PMF calculations. Data for each window was truncated from the beginning of the simulation in 5% increments until the final 50% of data remained (sequentially colored from red to blue). (TIF) [file pcbi.1004811.s007.tif]

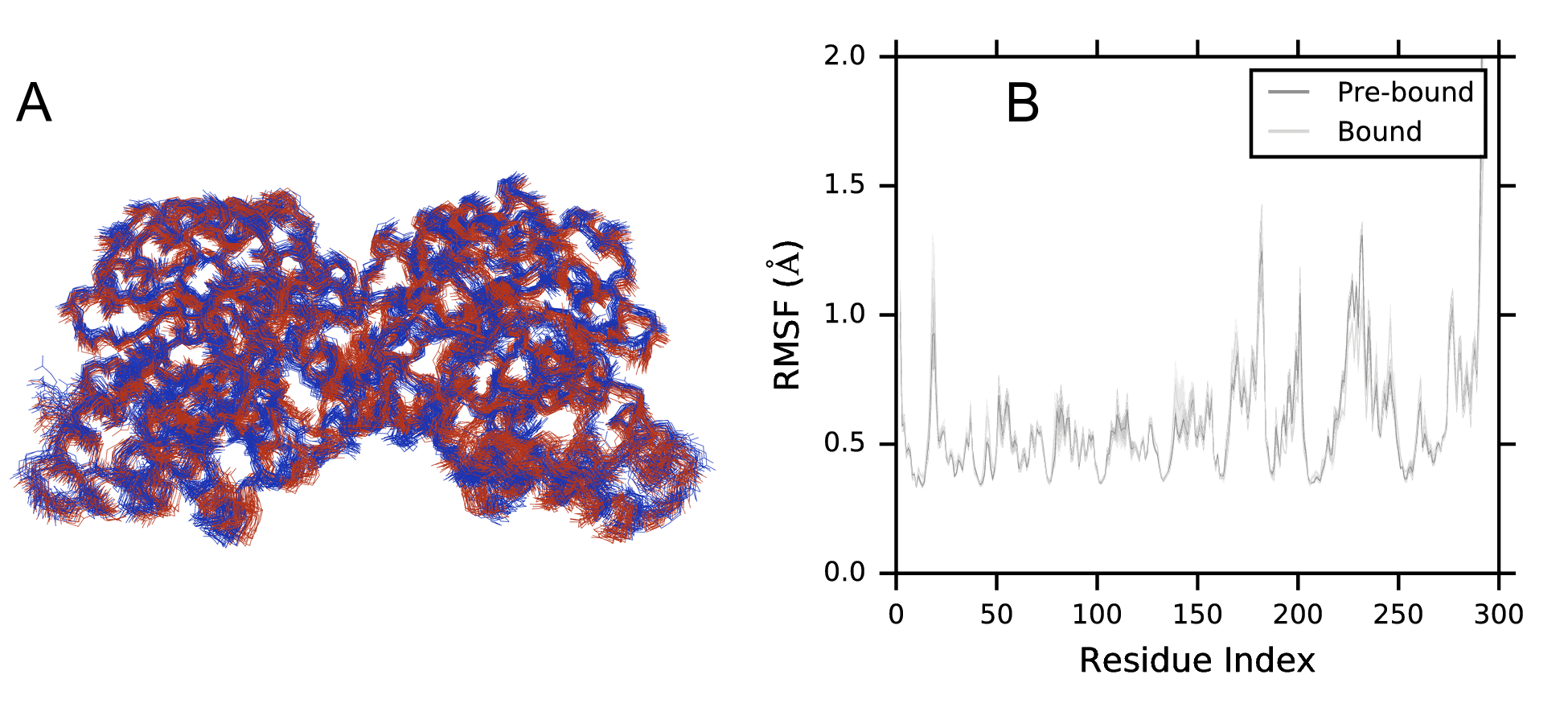

Supplement: S7 Fig — (A) Overlay of protein backbone atoms for the first 10 ns (red) and final 10 ns (blue) of a pyruvate binding simulation (100 ns). Pyruvate achieved an RMSD to the crystal structure (PDB ID 3DI1) [33] of <2.3 Å after approximately 16 ns, remaining bound until the end of the simulation. Snapshots were taken at 1 ns intervals. For reference, the complete simulation is provided in S1 Video. (B) root-mean square fluctuation (RMSF) analysis of DHDPS residues over the first 10 ns (pre-bound; dark gray) and the final 10 ns (bound; light grey) from several pyruvate binding simulations (mean ± standard error of the mean (SEM), n = 4). Values were calculated only for protein chain B. (TIF) [file pcbi.1004811.s008.tif]

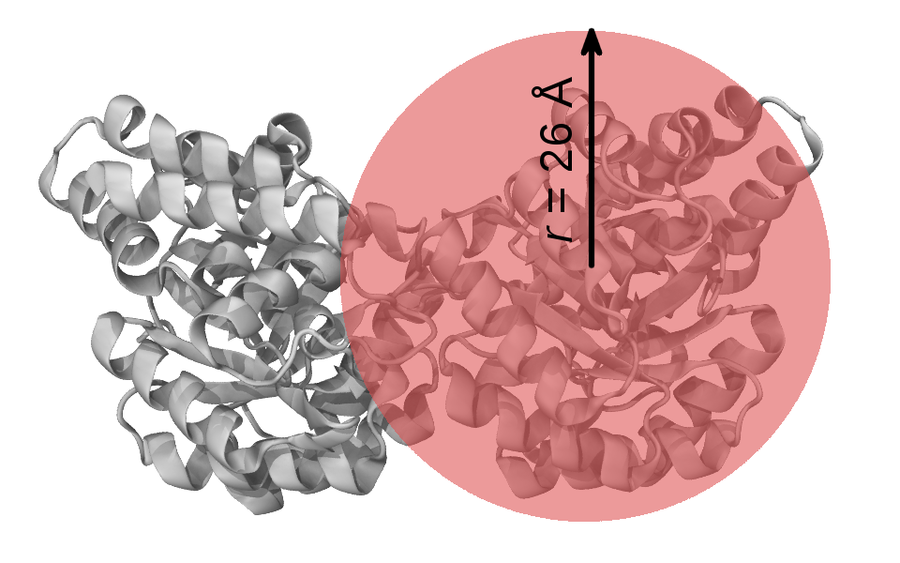

Supplement: S8 Fig — An upper limit restraint (26 Å) was placed upon the carboxylate carbon atom of pyruvate to restrict the searchable space available to the ligand. This restraint was relative to the position of the ζ-nitrogen of Lys163 from a single protein monomer with which pyruvate forms a Schiff-base during catalysis. (TIF) [file pcbi.1004811.s009.tif]
