# Supplementary material for: Dynamic Modelling Reveals ‘Hotspots’ on the Pathway to Enzyme-Substrate Complex Formation
Source: PLoS Comput Biol. 2016 Mar 11;12(3):e1004811. doi: 10.1371/journal.pcbi.1004811 (PMC4788353; doi:10.1371/journal.pcbi.1004811)
Supplement: S1 Table — Each window was simulated a total of 5 ns (25050 points). Only the final 2.5 ns were used for WHAM analyses and autocorrelation. (PDF) [file pcbi.1004811.s010.pdf]

# Dynamic Modelling Reveals 'Hotspots' on the Pathway to Enzyme-Substrate Complex Formation

Shane E. Gordon[1,2], Daniel K. Weber[2], Matthew T. Downton[2], John Wagner[2], Matthew A. Perugini[1,*]

**1 Department of Biochemistry and Genetics, La Trobe Institute for Molecular Science, La Trobe University, Melbourne, VIC 3086, Australia**
**2 Computational Sciences, IBM Research - Australia · Level 5 · 204 Lygon Street · Carlton VIC 3053**

*** M.Perugini@latrobe.edu.au**

## SI Table 1

**SI Table 1. Summary of weighted histogram analysis method (WHAM) of umbrella sampling windows. Each window was simulated a total of 5 ns (25050 points). Only the final 2.5 ns were used for WHAM analyses and autocorrelation. Free energy errors were attributed using 100 Monte Carlo bootstrap trials as described in Methods.**

| | Center (Å) | Spring Constant (kcal mol$^{-1}$ Å$^{-2}$) | Free Energy (kcal mol$^{-1}$ Å$^{-1}$) | Time Correlation (points) |
|---|---|---|---|---|
| 1 | 5 | 7.5 | $0 \pm 0$ | 2 |
| 2 | 6 | 7.5 | $-2.55 \pm 0.02$ | 2 |
| 3 | 7 | 7.5 | $-2.99 \pm 0.04$ | 122 |
| 4 | 7.5 | 10 | $-2.95 \pm 0.07$ | 22 |
| 5 | 8 | 10 | $-4.42 \pm 0.07$ | 4 |
| 6 | 8.5 | 10 | $-5.77 \pm 0.08$ | 4 |
| 7 | 9 | 10 | $-6.92 \pm 0.08$ | 2 |
| 8 | 9.5 | 10 | $-8.11 \pm 0.09$ | 7 |
| 9 | 10 | 7.5 | $-9.34 \pm 0.09$ | 950 |
| 10 | 11 | 7.5 | $-14.51 \pm 0.09$ | 8 |
| 11 | 12 | 7.5 | $-17.49 \pm 0.09$ | 8 |
| 12 | 13 | 7.5 | $-17.70 \pm 0.10$ | 7 |
| 13 | 13.5 | 10 | $-16.62 \pm 0.11$ | 11 |
| 14 | 14 | 10 | $-14.92 \pm 0.11$ | 483 |
| 15 | 14.5 | 10 | $-13.48 \pm 0.12$ | 17 |
| 16 | 15 | 7.5 | $-12.75 \pm 0.11$ | 11 |
| 17 | 16 | 7.5 | $-11.13 \pm 0.12$ | 4 |
| 18 | 16.5 | 10 | $-9.74 \pm 0.13$ | 5 |
| 19 | 17 | 7.5 | $-9.34 \pm 0.13$ | 29 |
| 20 | 18 | 7.5 | $-9.34 \pm 0.14$ | 141 |
| 21 | 18.5 | 10 | $-9.22 \pm 0.15$ | 10 |
| 22 | 19 | 10 | $-8.93 \pm 0.16$ | 23 |
| 23 | 19.5 | 10 | $-8.77 \pm 0.16$ | 5 |
| 24 | 20 | 7.5 | $-9.19 \pm 0.16$ | 7 |
| 25 | 21 | 7.5 | $-8.93 \pm 0.16$ | 21 |
| 26 | 22 | 7.5 | $-8.03 \pm 0.16$ | 58 |
| 27 | 23 | 7.5 | $-7.89 \pm 0.16$ | 35 |
| 28 | 24 | 7.5 | $-7.78 \pm 0.16$ | 21 |
