# Supplementary material for: Dynamic Modelling Reveals ‘Hotspots’ on the Pathway to Enzyme-Substrate Complex Formation
Source: PLoS Comput Biol. 2016 Mar 11;12(3):e1004811. doi: 10.1371/journal.pcbi.1004811 (PMC4788353; doi:10.1371/journal.pcbi.1004811)
Supplement: S2 Table — (PDF) [file pcbi.1004811.s011.pdf]

Dynamic Modelling Reveals 'Hotspots' on the Pathway to Enzyme-Substrate Complex Formation

Shane E. Gordon[1,2], Daniel K. Weber[2], Matthew T. Downton[2], John Wagner[2], Matthew A. Perugini[1,*]

1 Department of Biochemistry and Genetics, La Trobe Institute for Molecular Science, La Trobe University, Melbourne, VIC 3086, Australia
2 Computational Sciences, IBM Research - Australia · Level 5 · 204 Lygon Street · Carlton VIC 3053

*** M.Perugini@latrobe.edu.au**

SI Table 2

SI Table 2. Structural deviations between apo and pyruvate-bound forms of dihydrodipicolinate synthase.

	A/B[a]	PDB ID	Resolution (Å)	RMSD (Å)[b]
S. aureus	A	3DAQ [1]	1.45	0.19
	B	3DI1 [2]	2.20	
E. coli	A	1YXC [3]	1.90	0.12
	B	3DU0 [4]	2.00	
C. botulinum	A	3IRD	2.23	0.18
	B	3A5F	1.19	
A. tumefaciens	A	4I7U [5]	1.55	0.13
	B	4I7V [5]	1.45	

[a]A, apo; B, bound
[b]Calculations performed using backbone non-hydrogen atoms, chain A only.

References

1. Burgess BR, Dobson RCJ, Bailey MF, Atkinson SC, Griffin MDW, Jameson GB, et al. Structure and evolution of a novel dimeric enzyme from a clinically important bacterial pathogen. J Biol Chem. 2008;283(41):27598–603.

2. Girish TS, Sharma E, Gopal B. Structural and functional characterization of *Staphylococcus aureus* dihydrodipicolinate synthase. FEBS Lett. 2008;582(19):2923–30.

3. Dobson RCJ, Griffin MDW, Jameson GB, Gerrard JA. The crystal structures of native and *(S)*-lysine-bound dihydrodipicolinate synthase from *Escherichia coli* with improved resolution show new features of biological significance. Acta Crystallogr Sect D Biol Crystallogr. 2005;61(8):1116–24.

4. Devenish SRA, Gerrard JA, Jameson GB, Dobson RCJ. The high-resolution structure of dihydrodipicolinate synthase from *Escherichia coli* bound to its first substrate, pyruvate. Acta Crystallogr F Struct Biol Crystalliz Comm. 2008;64(12):1092–5.

5. Atkinson SC, Hor L, Dogovski C, Dobson RCJ, Perugini MA. Identification of the bona fide DHDPS from a common plant pathogen. Proteins: Struct, Funct, Bioinf. 2014;82(9):1869–83.
