# Supplementary material for: Dynamic Modelling Reveals ‘Hotspots’ on the Pathway to Enzyme-Substrate Complex Formation
Source: PLoS Comput Biol. 2016 Mar 11;12(3):e1004811. doi: 10.1371/journal.pcbi.1004811 (PMC4788353; doi:10.1371/journal.pcbi.1004811)
Supplement: S1 Text — (PDF) [file pcbi.1004811.s012.pdf]

Dynamic Modelling Reveals 'Hotspots' on the Pathway to Enzyme-Substrate Complex Formation

Shane E. Gordon[1,2], Daniel K. Weber[2], Matthew T. Downton[2], John Wagner[2], Matthew A. Perugini[1,*]

1 Department of Biochemistry and Genetics, La Trobe Institute for Molecular Science, La Trobe University, Melbourne, VIC 3086, Australia
2 Computational Sciences, IBM Research - Australia · Level 5 · 204 Lygon Street · Carlton VIC 3053

*** M.Perugini@latrobe.edu.au**

SI Text 1

Validating the MSM

A common check for Markovian behaviour ('Markoviany') of a particular model is to examine the lag time dependence of relaxation time-scales [1]. From the transition probability matrix T, we can establish the lag time dependence of relaxation time-scales in our model using the relationship:

$$\kappa_\tau = -\frac{\tau}{\ln \lambda_i(\tau)} \tag{1}$$

where κ is the relaxation time of eigenvalue λ_i of the transition probability matrix using a lag time of τ. At appropriately large values of τ, relaxation time-scales of eigenvalues become relatively insensitive to increases in lag time, satisfying the requirements of Markoviany [1]. The lag time cut-off beyond which implied time-scales plateau is termed the 'Markov time'.

Here, implied time-scales were calculated in the range of 0–15 ns at intervals of 0.25 ns (S2 Fig). The 10 slowest implied time-scales were found to plateau at 3 ns, indicating that the system is Markovian beyond this lag time. Thus, we constructed our Markov state model (MSM) using a lag time of 4 ns.

References

1. Prinz JH, Wu H, Sarich M, Keller B, Senne M, Held M, et al. Markov models of molecular kinetics: generation and validation. J Chem Phys. 2011;134(17):174105.
